# Could Alerting Physicians for Low Alkaline Phosphatase Levels Be Helpful in Early Diagnosis of Hypophosphatasia?

**DOI:** 10.4274/jcrpe.4426

**Published:** 2018-02-26

**Authors:** Asma Deeb, Abubaker Elfatih

**Affiliations:** 1 Mafraq Hospital, Clinic of Paediatric Endocrinology, Abu Dhabi, United Arab Emirates; 2 Shaikh Khalifa Medical City, Clinic of Biochemistry, Abu Dhabi, United Arab Emirates

**Keywords:** Alkaline phosphatase, hypophosphatasia, inborn error, laboratory, biochemistry

## Abstract

**Objective:**

Hypophosphatasia (HPP) is an inborn error of metabolism with significant morbidity and mortality. Its presentation is nonspecific leading to delayed or missed diagnosis. Low alkaline phosphatase (ALP) is a diagnostic test. Unlike high ALP, low level is commonly not flagged by laboratories as abnormal. A new treatment was shown to be effective in HPP. In this study we aimed to establish the frequency of low ALP levels requiring notification to physicians by the laboratory and also to describe the clinical manifestations of patients presenting with low ALP for a possible diagnosis of HPP.

**Methods:**

Patients under age 18 years with low ALP levels were identified from biochemistry records over a period of 6 months. Reference ranges were used as per the Associated Regional and University Pathologists Reference Laboratory (Utah, USA). Electronic results for patients with low levels were checked for flagging as abnormal/low ALP results. Charts of identified patients were reviewed. Presenting features were categorized under groups of disorders.

**Results:**

ALP levels were tested in 2890 patients. 702 had values less than 160 U/L. Of these patients, 226 (32%) had age/gender specific low ALP. None of the low ALP results was flagged as low. Twenty-one had more than one low reading and their charts were reviewed. Four patients in the neuromuscular and four in the miscellaneous group presented with features consistent with HPP despite these patients having no specific diagnoses.

**Conclusion:**

Laboratories do not alert physicians in cases with low ALP levels. A persistently low level in patients with unspecified diagnoses could be a key to diagnose HPP. Implementing lab-specific ranges and alerting for low levels could prompt physicians to investigate for undiagnosed HPP.

## What is already known on this topic?

Hypophosphatasia is a rare disorder with significant morbidity and mortality. A high level of alkaline phosphatase is commonly highlighted by biochemistry labs. Asfotase alpha is a new and effective medication for hypophosphatasia treatment.

## 

### What this study adds?

Unlike high alkaline phosphatase, low alkaline phosphatase is not always highlighted by biochemistry labs.

Identification of children presenting with non-specific clinical features and who have more than one reading of low alkaline phosphatase could help diagnose children with hypophosphatasia. Devising lab specific reference ranges for alkaline phosphatase is important to avoid missing abnormally low levels.

## Introduction

Hypophosphatasia (HPP) is an inborn error of metabolism characterized by a low serum alkaline phosphatase (ALP) level due to a defect in the gene encoding the tissue-nonspecific isozyme of ALP (*TNSALP*) (1). Inheritance can be autosomal recessive or dominant. As many as 260 genetic mutations in the* TNSALP *gene have been associated with HPP (2). Penetrance is variable which results in a wide range of clinical features, with the spectrum ranging from stillbirth with no bone mineralization to early loss of teeth without bone symptoms. Clinically, there are six forms of HPP based mainly on age at presentation: perinatal (lethal); perinatal (benign); infantile; childhood; adult and odontohypophosphatasia (1,3). Severe forms of HPP (perinatal and infantile) are inherited as autosomal recessive traits and in milder forms (adult and odontohypophosphatasia), autosomal recessive and autosomal dominant inheritance coexist (4). Genotype is known to be associated with specific outcomes in the perinatal lethal type, whereas genotype/phenotype correlation is less pronounced in other, less severe forms (5). HPP causes major morbidity in patients with substantial bone disease, myopathy and weakness. Hypercalcemia associated with nephrocalcinosis is a known feature of HPP (1). Craniosynostosis and skull dysmorphology occur in around 40% of infants (6). HPP is almost always fatal early in life when severe skeletal disease is obvious at birth (1,7). Skeletal deterioration typically results in death from respiratory insufficiency (7). Bone fragility and recurrent fractures can be presenting features of HPP in childhood (8). The perinatal form might present with intractable seizures caused by secondary pyridoxine-deficiency encephalopathy. This is due to deficiency of ALP that is required for the metabolism of pyridoxal-5’-phosphate neurotransmitters (9). Accordingly, HPP should be considered in neonates presenting with convulsions responding to pyridoxine. Although some of the above features might point to the diagnosis of HPP, other presenting features of HPP can be less specific and include various symptoms and signs encountered in more common diseases (10). Accordingly, clinical diagnostic criteria for HPP are unspecific and confirming the diagnosis requires biochemical, radiological and possibly genetic testing. This fact has been a major reason for the disease to be both underdiagnosed and misdiagnosed (11). A low ALP is a key for differentiating the diagnosis of HPP from many other more common paediatric disorders (2). Alerts by biochemistry laboratories on abnormal levels of ALP are useful to draw attention to specific diagnoses. Although a high level of ALP is usually highlighted by biochemistry labs, low levels are not usually flagged. Alerting for low ALP level could be an opportunity for the early diagnosis of HPP patients presenting with nonspecific manifestations. Early detection of HPP will offer these patients the opportunity to benefit from a new enzyme replacement treatment that has recently been shown to be an effective modality to treat this potentially fatal disease (12,13). This study was designed to check if biochemistry laboratories alert physicians to low ALP levels and also on the necessity of examining clinical features in those patients who have persistently low ALP levels.

## Methods

Using a cut-off level of 160 U/L, the electronic records of the Biochemistry Laboratory at Mafraq Hospital, covering a study period of six months, from July 2014 to Dec 2014, were screened for patients aged 18 years or under who had low ALP readings (phase I). As the study was based on charts review, no consent was deemed necessary as per the local research and ethics committee who approved the study. The cut-off value of 160 U/L was selected based on the Associated Regional and University Pathologists (ARUP) Reference Laboratory (Utah, USA) online test directory, being the highest level of the low range of ALP (14) (www.aruplab.com). The list of patients with low ALP was filtered by age and gender in accordance with the ARUP lab reference ranges (phase II). The biochemistry laboratory records were also evaluated for highlighting abnormally. Patients who had at least two readings of ALP lower than normal value per age and gender together with no other normal values had their charts reviewed (phase III). 

Three groups of patients were excluded:

- Patients who had two low values of ALP but had one or more normal value detected on subsequent testing.

- Those with a single low value of ALP with a normal value after or before.

- Only one borderline normal value at one presentation of acute illness with no further history of illness. 

In the remaining records, a list of the main diagnoses of the patients was made and stratified into subcategories (phase IV). Details of the patients’ presentations, working diagnoses and features suspicious of HPP were noted. The categories of diseases linked with a possible diagnosis of HPP included musculo-skeletal, rheumatological, neurological, renal, respiratory-related diseases and fractures. The approximate number of patients with suspicion of HPP was estimated and their presenting features reported for each disease category was noted for use in further studies. The study was approved by the Research and Ethics Committee at Mafraq Hospital (approval number: MAF-REC-12/2015_06). The ALP level was measured on a fully automated Roche Cobas**^®^** 8000 modular analyzer series c701 system (Roche Diagnostics GmbH, Mannheim, Germany, 2010). The assay is a basic, standardized, colorimetric assay traceable to the International Federation of Clinical Chemistry Reference Gen2 method as an optimized assay. ALP is measured in a reaction whereby ALP catalyzes the cleavage of phosphate from 4-nitrophenyl phosphate to form 4-nitrophenoxide (benzenoid form), which undergoes spontaneous rearrangement at alkaline pH to the quinonoid form (yellow color). The reaction is followed by measuring absorbance of the reactant color at 405 nm on the automated analyzer detection system. The ALP assay performance specifications include an analytical measuring range of 5-1200 U/L, with a lower detection limit (analytical sensitivity ie the lowest measurable analyte level that can be distinguished from zero) of 5.00 U/L. The assay has a clinically reportable range of 5.00-6000 U/L. The ALP assay has a within-run precision coefficient of variation (CV%) of 0.7% at an ALP mean of 84.3 U/L and of 2.4% at a mean of 92.8 U/L, while the assay demonstrates a CV of 0.5% at an ALP mean of 222 U/L and a CV% of 1.7% at a mean of 224 U/L. The inter-individual CV is 6.7%, with an intra-individual CV of 25.4% and a critical significant difference of 37%.

## Results

**Figure 1:**
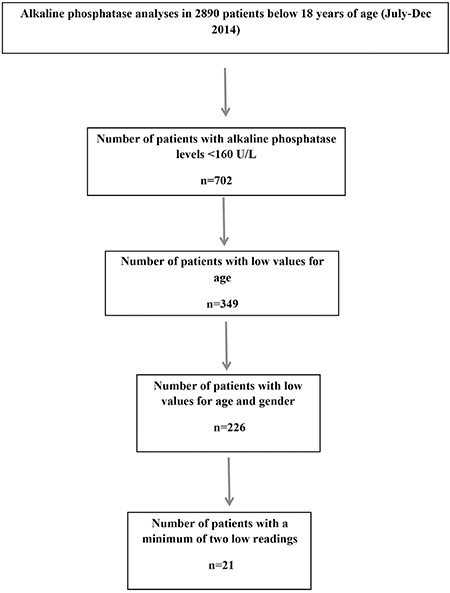
Flow chart showing of number subjects with low alkaline phosphatase levels by age and gender

During the six-month study period, there were 2890 tests for ALP performed in subjects 18 years of age or younger. In phase I of the study, this number was reduced to 702 patients who had readings below 160 U/L. None of the low levels of ALP was flagged as abnormal by the biochemistry lab. Age stratification for normal reference values was performed, resulting in 349 patients being selected (phase II). Further filtering was done to this group to match reference range with gender. This reduced the number of patients to 226 at this stage (phase III). Of those, 1 patient (male) was in the age bracket of 16-18 years, 24 (20 males) between 14-15 years, 24 (14 males) between 12-13, 19 between 10-11 years (NB from this age and younger, there is no gender difference in the quoted lower normal value for ALP), 35 between 7-9 years, 74 between 4-6 years, 48 between 1-3 years and 1 between 1-11 months (Table 1). Charts for all patients identified in phase III were reviewed. Two hundred and five patients were excluded as per the exclusion criteria and 21 patients were studied further (Figure 1). The 21 patients were classified under disease categories based on presentation and working diagnoses. These were; rheumatologic disorders (five patients), fractures (five patients), neuromuscular diseases (four patients), immobility and repeated fractures (three patients) and a miscellaneous group (four patients) (Table 2). The five patients in the rheumatology category had a confirmed diagnosis of systemic lupus erythematosus (three patients) and juvenile rheumatoid arthritis (two patients). Five patients had a single fracture of a long bone. Of those, one had a dislocated shoulder with a fracture of the humerus and another had orthodontic treatment for teeth malposition and crowding. Three patients were diagnosed with cerebral palsy and were immobile with repeated fractures. The neuromuscular category included four children who did not have definite diagnoses. Two had arthrogryposis, one of whom also had repeated fractures. One presented with multiple skeletal deformities and the fourth patient had neuromuscular deformities with fractures. The miscellaneous group included a child with Down syndrome who was diagnosed with short limbs antenatally and admitted to the intensive care unit repeatedly with recurrent chest infections. One child was diagnosed with nemaline myopathy and had kidney stones and another was diagnosed with severe demyelinating sensory and motor neuropathy. The fourth patient had repeatedly low ALP readings and suffered from recurrent infections. The selected patients could potentially have HPP as their presentation is quite unspecific and their ALP is persistently low. Further diagnostic testing (particularly genetic testing) is recommended in these scenarios. This is mentioned below as a limitation of our study.

## Discussion

HPP is a disease that is associated with major co-morbidity and poor prognosis. The wide range of presenting features which are non-specific constitutes a complicating factor in its diagnosis (11). In the past, various treatment approaches have been tried to treat the severe form of the disease with poor results. Treatment modalities included transplantation therapy using bone fragments and cultured osteoblasts (7), infusion of enriched plasma with ALP from patients with Paget disease (15), bone marrow transplant (16) and conservative treatment using low calcium milk and pamidronates (17). Calcitonin and chlorothiazide have been used to reduce calcium level, which can reach very high levels (18). Bisphosphanates are pyrophosphate analogs and can precipitate the disease progression. Patients with undiagnosed HPP presenting with fractures and osteoporosis and treated with bisphosphanate are reported to progress into renal failure (19) and using bisphosphanate to treat HPP is currently contraindicated. Asfotase Alfa is a recombinant, fusion protein comprising the *TNSALP* ectodomain and a terminal deca-aspartate motif for bone targeting (20). It has been used in clinical trials and was shown to enhance healing of skeletal abnormalities and improve respiratory and motor dysfunction (12). Asfotase alfa has now been approved by the European Medicines Agency for use in patients with HPP (13).

In our cohort, we detected a group of patients who had low ALP levels, but no specific diagnosis (Table 2). Despite the low ALP level in more than one occasion of testing, there was no alert by the biochemistry laboratory drawing attention to the low value. In two groups of patients, those with rheumatic diseases and those with fractures causing immobility, the ALP abnormality could be possibly attributed to the underlying disease. In a third group of patients, those with single fractures, the patients were healthy otherwise and unlikely to have an undiagnosed HPP. However, some patients in the neuromuscular disorder group (four patients) and the miscellaneous group (four patients) are worth examining further to rule out the possibility of HPP. Two patients in particular in the miscellaneous group had repeated episodes of chest infection and intensive care unit admissions and one had a kidney stone. The four patients in the neuromuscular group had skeletal deformities and fractures and they did not have a specific diagnosis. They, too, qualify for further investigations to exclude HPP. High levels of ALP can be encountered in a variety of bone disorders, but low levels are not as frequently seen in clinical practice. A high ALP level is routinely flagged up by the biochemistry lab but this is not the case for low ALP levels. Biochemistry labs need to have reference ranges for ALP levels to highlight possible abnormalities particularly in case of associated hypercalcemia. The ALP assay is widely available and is a fairly inexpensive test. It is a key for diagnosing HPP and makes a good screening test to diagnose HPP for which an effective treatment is now available.

### Study Limitations

The main limitation of the study is that we did not confirm the diagnosis of HPP in those suspected cases where a definite cause for the ALP was not reached. Further plans for genetic testing on such suspected cases will facilitate the diagnosis.

## Conclusion

We conclude that persistent low ALP levels in patients presenting with non-specific signs and symptoms can be used as a guide to further investigate and exclude HPP. This is particularly important because medication is now available for HPP and has been shown to be effective in ameliorating morbidity and improving quality of life in this disease. Accordingly, the alerting of physicians to low levels of ALP by biochemistry labs can be very useful. We highlight the importance of having age and gender adjusted ALP reference ranges, specific for local laboratories or populations, to avoid missing the diagnosis of HPP. A clear plan of action needs to be drawn on how to proceed with patients with low ALP levels and non-specific presentation.

## Figures and Tables

**Table 1:**
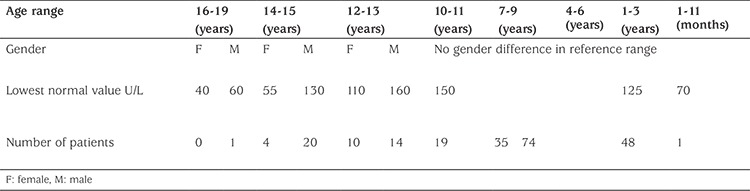
Numbers of patients with low alkaline phosphatase concentrations for age and gender (total 226)

**Table 2:**
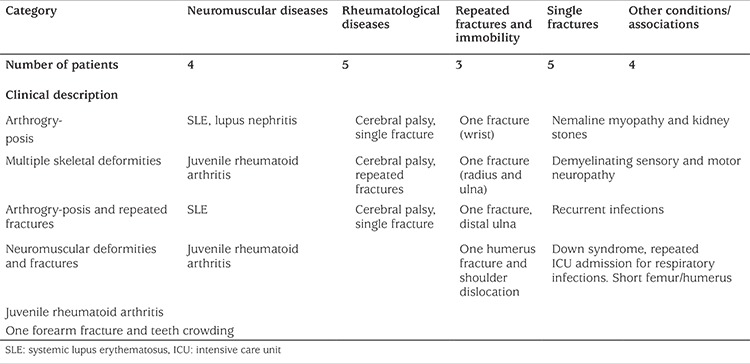
System involvement in 21 patients with persistent low alkaline phosphatase and repeated medical presentations
